# Tau Hyperphosphorylation and Oxidative Stress, a Critical Vicious Circle in Neurodegenerative Tauopathies?

**DOI:** 10.1155/2015/151979

**Published:** 2015-10-20

**Authors:** Seyedeh Maryam Alavi Naini, Nadia Soussi-Yanicostas

**Affiliations:** ^1^INSERM UMR 1141, Hôpital Robert Debré, Paris, France; ^2^Université Paris Diderot, Sorbonne Paris Cité, Paris, France

## Abstract

Hyperphosphorylation and aggregation of the microtubule-associated protein tau in brain, are pathological hallmarks of a large family of neurodegenerative disorders, named tauopathies, which include Alzheimer's disease. It has been shown that increased phosphorylation of tau destabilizes tau-microtubule interactions, leading to microtubule instability, transport defects along microtubules, and ultimately neuronal death. However, although mutations of the *MAPT* gene have been detected in familial early-onset tauopathies, causative events in the more frequent sporadic late-onset forms and relationships between tau hyperphosphorylation and neurodegeneration remain largely elusive. Oxidative stress is a further pathological hallmark of tauopathies, but its precise role in the disease process is poorly understood. Another open question is the source of reactive oxygen species, which induce oxidative stress in brain neurons. Mitochondria have been classically viewed as a major source for oxidative stress, but microglial cells were recently identified as reactive oxygen species producers in tauopathies. Here we review the complex relationships between tau pathology and oxidative stress, placing emphasis on (i) tau protein function, (ii) origin and consequences of reactive oxygen species production, and (iii) links between tau phosphorylation and oxidative stress. Further, we go on to discuss the hypothesis that tau hyperphosphorylation and oxidative stress are two key components of a vicious circle, crucial in neurodegenerative tauopathies.

## 1. Introduction

The tauopathies are a class of neurodegenerative disorders characterized by hyperphosphorylation and aggregation of the microtubule-associated protein tau (MAPT) into paired helical filaments (PHFs) or straight filaments (SFs), forming neurofibrillary tangles (NFTs) in brain. Unlike amyloid-beta (A*β*) aggregation, which is associated with Alzheimer's disease (AD), tau tangles are found in multiple neurodegenerative disorders such as progressive supranuclear palsy (PSP), corticobasal degeneration (CBD), Pick's disease, dementia pugilistica, frontotemporal dementia with parkinsonism linked to chromosome 17 (FTDP-17), and many other disorders including AD [1]. Mutations in the* MAPT* gene have been linked with several familial early-onset tauopathies [1]. More than 50 pathogenic mutations have been identified in the* MAPT* gene [1], providing evidence that tau alterations alone can cause neurodegeneration. It has been shown that abnormal tau hyperphosphorylation impairs its binding to microtubules and its capacity to promote microtubule assembly, resulting in its self-aggregation into NFTs, microtubule disorganization, and impaired transport along axonal microtubules [2, 3].

In addition to tau hyperphosphorylation, a growing body of evidence suggests that oxidative stress (OS) is another component of the pathophysiology of tauopathies. According to the OS theory of aging, which was deduced from genetic studies showing that manipulation of antioxidant defenses affects longevity in several animal models, brain neurons are seen as a crucial target of oxidative attacks. Moreover, OS has been implicated in the disease process in several neurodegenerative disorders, including AD [4]. In AD, the link between the production of toxic A*β* peptide and OS is well documented and has been the subject of several recent reviews [5, 6]. However, other data support an essential role for OS in tau hyperphosphorylation, tau polymerization, and tau toxicity. The accumulation of OS markers is now seen as a further hallmark of tau pathology in both patients and animal models. However, the precise role of OS in tauopathies remains far from clear.

Here, after a brief summary of our knowledge of tau structure and function, we review evidence suggesting that OS is both a late consequence of tau pathology paralleling the course of the disease, and an early cellular response to injuries linked to tau toxicity. Lastly we discuss the hypothesis that tau hyperphosphorylation and OS are the two key elements of a vicious circle, crucial in tau pathology.

## 2. Structure and Posttranslational Modifications of the Tau Protein

**Figure 1:**
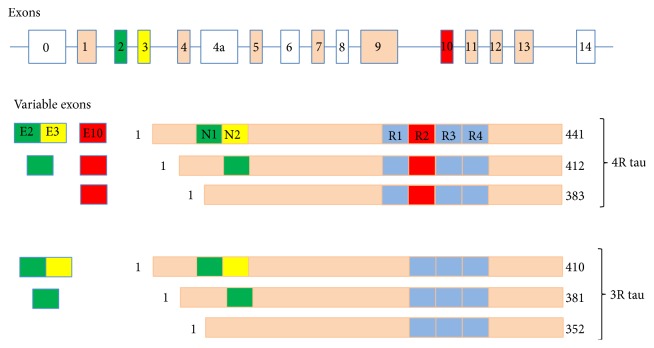
The* MAPT* gene, the variable exons, and the six tau isoforms in the adult human brain generated by alternative splicing. The constitutively spliced exons are shown in beige. E0, E4a, E6, E8, and E14 are not transcribed in human brain. Alternative mRNA splicing of E2 (green), E3 (yellow), and E10 (red) generates six tau isoforms ranging from 352 to 441 aminoacids. Three isoforms have four repeats each (4 repeat) and three isoforms have three repeats each (3 repeat). The repeats are shown with R (R1 to R4). The exons and introns are not shown to scale.

Tau is a highly soluble, natively unfolded, and phosphorylated protein predominantly located in axons of mature neurons [7–9]. Tau is also found in the neuronal somatodendritic compartment [10] and nucleus [11], and to a lesser extent in astrocytes and oligodendrocytes [12]. Six tau isoforms are expressed in the CNS in human adults [13]. The six isoforms are generated* via* alternative splicing of a single* MAPT* gene located at 17q21.31 and comprising 16 exons [14]. Tau isoforms range in length from 352 to 441 amino acids, and from 45 to 65 kDa, with exons 1, 4, 5, 7, 9, 11, 12, and 13, translated in all tau isoforms. Tau isoforms differ by the presence of three (3R) or four (4R) carboxy-terminal tandem repeat sequences of 31 amino acids, corresponding to microtubule-binding domains (MBDs). Tau isoforms also differ by the absence (0N) or presence of one (1N) or two (2N) N-terminal repeated sequences encoded by alternative exons 2 and 3. The 3R- and 4R-tau isoforms are found in equal amounts in the adult human brain; while the 0N, 1N, and 2N tau isoforms comprise about 37%, 54%, and 9% of total tau, respectively [15, 16] (Figure 1).

The tau protein found in the PNS is named “big tau,” in reference to its high molecular weight compared with isoforms expressed in CNS neurons. This higher weight stems from the inclusion of exon 4a in the amino-terminal half of the protein [17]. Expression of different tau isoforms is tissue-specific [13] and dynamic during development, probably owing to its key role in cytoskeletal plasticity during embryogenesis and early development. In humans, only the shortest tau isoform (3R/0N) is expressed in fetal brain, whereas all six isoforms are found in adult CNS [18].

Posttranslational modifications are a major factor behind the diversity of tau. Besides phosphorylation, the crucial posttranslational modification in tau, this protein also undergoes an array of other posttranslational changes, such as* O*-glycosylation [19], ubiquitination [20], SUMOylation [21], nitration [22], glycation [23], acetylation [24], cross-linking by transglutaminase [25], isomerization [26], conformational alteration, and proteolytic cleavage [27], all of which are involved in tau regulation and pathology.* O*-GlcNAcylation (a form of tau* O*-glycosylation) reduced tau phosphorylation in cerebral cortex and hippocampus in rat [28], and in Alzheimer's disease,* O*-GlcNAcylation levels negatively correlate with tau phosphorylation [29]. Tau acetylation at lysine residue 280 increased tau fibrillization and decreased tau-dependent microtubule assembly* in vitro*. Tau acetylation was also found to be strongly associated with tau hyperphosphorylation and tau inclusions in Alzheimer's disease, corticobasal degeneration, progressive supranuclear palsy, and in tau transgenic mouse models of tauopathies [24, 30]. However, the precise functions of tau modifications remain enigmatic.

## 3. Functions of the Tau Protein

The main function recognized for tau is promoting microtubule polymerization and stabilization. Microtubules form part of the cytoskeletal framework in all eukaryotes and are composed mainly of heterodimers of *α*- and *β*-tubulin forming tubular polymers. Microtubules play a major role in cytoskeletal maintenance and act as highways for intracellular transport of organelles, vesicles, proteins, and signaling molecules [31].

The 4R-tau isoform displays the highest affinity for microtubules and efficiently promotes microtubule assembly, likely due to the presence of the interrepeat sequence located between the first and second MBDs [16]. Binding of tau to microtubules has been visualized by NMR spectroscopy [32] and cryo-EM [33]. However, some studies have challenged the primary function of tau as main regulator of microtubule stability and assembly. In cell culture, tau colocalizes with the most dynamic microtubules [9], those showing the highest basal turnover rate among microtubule populations [34]. Moreover, siRNA-mediated knockdown of tau does not impair microtubule polymerization [35]. In addition, tau,* via* its MBD, interacts with other proteins such as actin [36], presenilin-1 (PS-1) [37], tau phosphatases [38], RNA [39], and DNA [40], strongly suggesting that tau has multiple functions apart from its putative role as modulator of microtubule dynamics.

The N-terminal domain of tau interacts with Src homology 3 (SH3) domains in a number of signaling proteins [41], such as Fyn tyrosine kinase. Tau also promotes process extension in oligodendrocytes by connecting Fyn to microtubules [12]. Tau also stimulates Src-mediated actin rearrangements after growth factor stimulation [42]. Interestingly, the binding of tau to neuronal plasma membrane components and SH3 domains is affected by its phosphorylation [43, 44]. Other functions of tau include binding and activation of phospholipase C (PLC) *γ* [45], inhibition of the enzyme histone deacetylase-6 (HDAC-6) [46], modulation of the cellular response to heat shock [47], and adult neurogenesis [48]. Lastly, it was recently shown that the 2N/4R tau isoform plays a role in microglial activation in humans [49]. These data suggest that tau is a scaffolding protein able to (i) bind to at least two signaling proteins, (ii) restrict signaling molecules and transduction pathways to defined cellular compartments, and (iii) regulate several signaling pathways [50].

Notwithstanding these multiple hypothetical functions, the relationship between tau and microtubules is well established. In particular, tau appears to modulate axonal transport* via* interaction with motor proteins, such as detachment of kinesin from microtubules and directional reversal of dynein, without affecting the speed of transport [51–53]. High levels of tau accumulation are detected in the axonal region near the synapse [54], which may facilitate cargo delivery to presynaptic terminals [51]. Moreover, while several observations suggest that removal or overexpression of tau* in vitro* or* in vivo* does not impair axonal transport [55, 56], other studies suggest that overexpression of wild-type or mutant tau in either cell or mice models of tauopathy impairs axonal transport [57, 58]. These discrepancies may be due to differences in study design or model systems used.

Four tau knockout (KO) lines have been independently generated in mice [59–62]. All four lines are viable and fertile, with no apparent phenotypes. Harada et al. [59] generated the first mouse tau KO strain. Apart from the absence of an overt phenotype, these mice showed reduced microtubule stability and mild disorganization of small-caliber axons. In another tau KO line, Dawson et al. [60] reported a delay in neuronal maturation in* in vitro* culture from the tau KO line. However, in both KO lines, microtubule-associated protein (MAP) 1A was found to be elevated during early developmental stages, suggesting that overexpression of MAP1A is able to compensate for the loss of tau. Interestingly, an increased accumulation of MAP1A was also reported in the third tau KO line, which was generated by Fujio et al. [62]. In addition, despite normal levels of MAP1B in tau KO mice generated by Harada et al. [59], cross-breeding of this line with MAP1B-deficient mice resulted in cerebral developmental defects and early lethality [63], suggesting a further functional overlap between tau and this MAP.

Precise investigations of tau KO mice generated by Harada et al. [59] revealed progressive behavioral impairments and motor deficits in older individuals. These phenotypes comprised muscle weakness, impaired balance, hyperactivity, and learning deficits from 12 months of age onward [64]. More recently, complex motor deficits linked to dopaminergic neuronal loss and increased iron accumulation in neurons in substantia nigra were reported in another tau KO line [65]. Tau KO mice also showed deficits in neuronal circuit formation as revealed by electrophysiology [66]; alongside these negative consequences, tau KO mice are more resistant to epileptogenesis, excitotoxicity, and beta-amyloid toxicity [67, 68].

Tau overexpression in neuronal cell cultures results in an increased number of neurites per cell [69]. Moreover, while insertion of human* MAPT* gene in mice leading to expression of all six tau isoforms does not induce severe neuropathology [70], expression of this transgene in a mouse tau KO context resulted in tau hyperphosphorylation and accumulation of insoluble 3R tau [71]. These mice also develop neurodegeneration and age-dependent memory loss [72, 73]. By contrast, hind-limb clasping and spinal cord abnormalities accompanied with somatodendritic distribution of the human tau protein were detected in human 2N4R tau cDNA mouse, while endogenous mouse tau was present [70]. A transgenic line with KI insertion of human 2N4R tau cDNA in the first exon of the mouse* MAPT* gene displayed increased neuronal survival accompanied by better performance in a novel object recognition task [74]. Apart from different types of transgenes and the presence or absence of endogenous mouse tau yielding different phenotypes, the promoter type also profoundly affected the observed phenotypes. Severalfold overexpression of the shortest human tau isoform to endogenous mouse tau resulted in axonal degeneration and progressive motor weakness [75].

The precise mechanism of hyperphosphorylated tau-mediated neurotoxicity remains to be elucidated, but two major mechanisms have emerged to date: (i) toxic loss of function, with loss of physiological tau function held responsible for tau pathology [76–78] and (ii) toxic gain of function with highly phosphorylated tau displaying ill-defined toxic effects in neuronal cells [72, 79, 80]. Interestingly, it was shown that hyperphosphorylated tau relocates to dendritic spines, where it may exert its toxic effect by impairing trafficking and/or synaptic anchoring of glutamate receptors [81].

More than 50 mutations of the* MAPT* gene have been identified to date that are associated with neurodegenerative tauopathies [1].  Table 1 lists the most important pathogenic tau mutations. Many of these mutations are missense mutations or small deletions, which modify tau sequence. In particular, several missense mutations such as G272V, P301L, P301S, V337M, G389R, and R406W decrease the* in vitro* affinity of tau to microtubules, resulting in deficits in microtubule assembly and stability [15, 82, 83]. By contrast, missense mutations S305N and Q336R slightly increase the ability of tau to promote microtubule assembly [84, 85]. In addition, most of these missense mutations enhance tau aggregation [86, 87]. Lastly, several mutations impair binding of tau to protein phosphatase 2A, one of the major tau phosphatases in brain neurons [88]. Other mutations are located in either intronic sequences close to the 5′ splice site of exon 10 (positions +3, +11, +12, +13, +14, +16, +19, and +29) or exonic sequences, also impairing exon 10 splicing (N279K, ΔK280, L284L, N296H, ΔN296, P301L, P301S, G303V, and S305N). All these mutations disrupt the physiological 1 : 1 ratio of 4R to 3R tau isoform in the adult human brain. In turn, the increased production of 4R tau leads to its assembly onto NFTs and ultimately neurodegeneration [89]. Other mutations in intronic sequences adjacent to the stem-loop structure in exon 10 increase accumulation of soluble 3R tau isoform through alteration of* MAPT* gene splicing. Interestingly, these mutations lead to neuronal apoptosis and increased accumulation of tau degradation products, but not the formation of NFTs [90]. Finally, several pathologic tau mutations are located in exon 10, such as ΔK280, ΔN296, and N296H, and induce tau protein accumulation and affect* MAPT* RNA levels [83, 91–93].

Although these results point to tau as a key factor essential for proper microtubule assembly and dynamics, they also highlight the complex and still poorly understood function of this protein.

## 4. Phosphorylation, Oligomerization, Aggregation, and Propagation of the Tau Protein

The activity of the phosphoprotein tau is mainly regulated by phosphorylation. Similar to the expression of different tau isoforms, tau phosphorylation is also developmentally regulated, which is important for cytoskeletal plasticity during early development. Tau phosphorylation also influences the structure, distribution, and function of the protein in neurons [99].

Tau phosphorylation is increased in both physiological and pathological contexts, but it is still unclear whether the mechanisms involved in physiological and pathological tau hyperphosphorylation overlap. Tau phosphorylation is markedly increased during embryonic development [100], likely due to the increased need for neuronal plasticity. Several cellular stress conditions such as OS [101–103], heat stress or hypothermia [103, 104], and even starvation [105] modulate tau phosphorylation. Hyperphosphorylated tau is also identified as the main component of NFTs [106]. High levels of hyperphosphorylated tau have been detected in the cerebrospinal fluid (CSF) of patients suffering from tauopathies. Levels of hyperphosphorylated tau in CSF also correlate with hippocampal atrophy in prodromal AD, also called “mild cognitive impairment” (MCI) [107, 108]. Tau is hyperphosphorylated in all tauopathies, but hyperphosphorylation states differ among and within disorders [109]. Importantly, no single phosphorylation site is specific for tauopathies, and hyperphosphorylation is characterized by an overall increase in tau phosphorylation at multiple residues. Also, tau hyperphosphorylation is defined as an increase in either the number of phosphorylated sites per tau molecule or the fraction of tau molecules phosphorylated at a given site. Thus 1.9 moles of phosphate per mole of tau is found in a healthy human brain, against 6 to 8 moles of phosphate per mole of tau in an AD brain [110]. However, the precise phosphorylation state of tau is difficult to define in* postmortem* biopsy material, due to the labile nature of phosphorylated tau, which quickly becomes dephosphorylated after excision [111].

**Figure 2:**
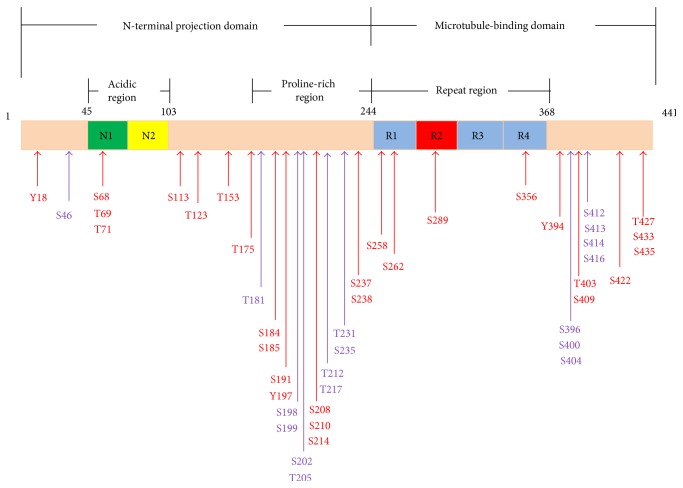
Schematic representation of the distribution of tau phosphorylation sites on the longest tau isoform (441 amino acids). The two amino-terminal inserts are demonstrated by E2 and E3. The microtubule-binding domains are represented with R(1–4). Physiologic tau phosphorylation comprises approximately 10 phosphorylated residues. The physiologically phosphorylated residues are shown in purple and cluster in the proline-rich domain (PRD) and in the C-terminal region. The number of phosphorylated residues rises to 45 on the longest tau isoform from Alzheimer brain with the appearance of phosphorylated residues shown in red. The two amino-terminal inserts and repeat regions are not physiologically phosphorylated in adult human brain. A number of phosphorylation sites are detected in both regions on the longest tau isoform from Alzheimer brain.

Nearly 45 phosphorylated sites have been identified on tau extracted from AD brain, that is, more than half of all the 85 theoretical phosphorylable residues on the longest tau isoform (2N4R) (Figure 2). This situation contrasts with the approximately 10 and 18 phosphorylated residues found on soluble tau extracted from adult human brain and fetal rat brain, respectively. Most hyperphosphorylated residues in AD are clustered in the C-terminal and the proline-rich domains of tau, and very few sites are located within the N-terminal region and MBD. Most phosphorylated sites have been identified by mass spectrometry or Edman degradation, and a few sites located mostly at the N-terminal region have been identified only by phosphospecific antibodies [112].

Tau hyperphosphorylation is thought to result from an imbalance in the function of several protein kinases and phosphatases [109]. While tau is phosphorylated by a large number of kinases* in vitro* [112], the identity of the kinases, which are responsible for physiological or pathological phosphorylation of tau* in vivo*, has so far remained elusive. In particular, as no single kinase is able to phosphorylate all pathological tau residues, several kinases may be involved in tau hyperphosphorylation. Tau kinases fall into the two main groups of proline-directed and non-proline-directed kinases. Proline-directed tau kinases GSK3*β* and CDK5 are important tau kinases that phosphorylate tau at a large number of serine and threonine residues and play an important role in tau pathology in AD [113, 114]. Furthermore, GSK3*β* activation and CDK5 overexpression were shown to induce tauopathy-related phenotypes in mouse models [115, 116]. Activity of nonreceptor tyrosine kinases (such as Fyn and c-Abl) have also been linked to AD pathology [117]. It has been proposed that tau phosphorylation by different kinases likely follows a sequential pattern, in which phosphorylation of given residues facilitates that of other phosphorylation sites. Such a sequential pattern of kinase activities has been suggested by* in vitro* observations of the effect of DYRK1A and CDK5 kinases on GSK3*β*-mediated tau phosphorylation [118, 119]. In addition, the activation of one kinase may activate a second one through a “kinase cascade,” as what was observed by the stimulatory effect of CK1 and c-Abl kinases on CDK5 [120, 121].

Phosphatases dephosphorylating tau* in vitro* include PP1, PP2A, PP2B, and PP5 [122]. PP2A displays the strongest dephosphorylating effect on tau [123] and may be an important regulator of tau phosphorylation* in vivo* [124]. A decrease in PP2A activity mediates hyperphosphorylation of tau in hypothermia [104]. Studies also suggest that PP2A expression and activity are markedly decreased in AD [125, 126]. Furthermore, an AD-like pathology has also been observed secondary to treatment with okadaic acid, a potent inhibitor of phosphatases 1 and 2A in rats [127, 128].

Increased tau phosphorylation decreased its affinity for microtubules: pathologic phosphorylation of tau at Ser396 and Ser404 decreases its binding affinity for microtubules [129]. Similarly, phosphorylation at S214 and T241 in the proline-rich domain reduces the binding of tau to microtubules [2].

Aggregation-prone tau species display toxicity in cell culture [130] and in a transgenic mouse model [131]. However, whereas it was previously thought that aggregated tau was toxic, more recent studies suggest that soluble and prefibrillar tau species are more likely to be implicated in neurodegenerative processes [132]. In particular, several observations suggest that tau oligomers, but not tau aggregates, are the toxic species. In AD patients, neuronal loss exceeds NFT number by at least one order of magnitude [133], and some familial tau mutations associated with frontotemporal dementia cause very few aggregated tau inclusions [134]. Tau oligomers and truncated tau species display toxicity* in vitro* [135, 136]. Following expression of the aggregation-prone tau^ΔK280^ construct in N2a neuroblastoma cells, the toxic tau species were identified as prefibrillar forms of tau before the *β*-sheet containing aggregates were detected [130]. Moreover, tau overexpression in several* Drosophila* models induces neurodegeneration without NFT formation [137, 138]. However, it must be borne in mind that the lifespan of* Drosophila* neurons is one order of magnitude lower than that of mammalian neuronal cells. Lastly, whereas accumulation of soluble forms of the tau protein correlates with neuronal and synaptic dysfunction and toxicity in several mouse models [80, 81, 139], in two different conditional mouse models of tauopathies, transgene silencing resulted in memory recovery while NFTs were still present [80, 139].

*In vitro*, recombinant full-length and nonphosphorylated tau is able to form filaments following interaction with negatively charged compounds such as sulfated glycosaminoglycans, RNA, or fatty acids [39, 140–142]. Moreover, both wild-type and mutant tau bearing G272V, ΔK280, P301L, P301S, S305N, V337M, or R406W mutations do not form filaments* in vitro* in the absence of heparin or other negatively charged compounds. However, the exact mechanism by which heparin induces formation of tau fibrils remains a matter of debate.

Interestingly, in a given tauopathy, tau aggregation propagates in a sequential and predictable fashion from one brain region to another, in a manner similar to that described for prion proteins. This stereotypical spatiotemporal spreading of tau aggregation has been described in AD [143] and argyrophilic grain disease (AGD) [144], suggesting that tau pathology spreads along defined neuronal pathways.* In vitro* studies have demonstrated intercellular transfer of tau inclusions in cultured cells [145]. Sulfated glycosaminoglycans at the cell surface are required for internalization of aggregated tau [146]. This phenomenon has also been observed* in vivo* in mice expressing wild-type human 4R tau isoform, following injection of brainstem extracts from mice expressing human tau^P301S^ [147]. Also, formation of tau inclusions is accelerated when filaments assembled from recombinant human tau^P301S^ were injected into the brains of young tau^P301S^ transgenic mice before the formation of tau aggregates [148]. In addition, transgenic mice overexpressing human tau^P301L^ in restricted areas of the entorhinal cortex and subiculum showed a propagation of the pathology in other synaptically connected brain regions [149, 150]. The data suggest that filamentous tau can convert soluble tau to fibrillar tau, forming tau inclusions.

## 5. Neurodegenerative Tauopathies

Tauopathies are heterogeneous disorders with partially overlapping clinical, neuropathological, and genetic characteristics, forming a spectrum of disorders. These diseases are characterized by microtubule-associated tau protein abnormalities [1]. Tau inclusions occur in neurons, astrocytes, and oligodendrocytes [151, 152].

Intraneuronal tau inclusions display different morphologic features in tauopathies, ranging from NFTs and neuropil threads to dystrophic neurites and Pick bodies. Tau aggregates are also differentiated by the phosphorylation and isoform content of tau, allowing a molecular classification of tauopathies. A biochemical classification based on the electrophoretic profile of tau discriminates four distinct classes [153].

Class I tauopathies are characterized by three major electrophoretic tau isoforms at 60, 64, and 69 kDa and a minor isoform at 72/74 kDa. This profile corresponds to the aggregation of the six tau isoforms in the human brain and characterizes AD, Down syndrome, and several other tauopathies. Class II forms are characterized by two major electrophoretic isoforms at 64 and 69 kDa and a minor band at 74 kDa. This profile characterizes several tauopathies such as CBD, AGD, and PSP. The profile of Class III tauopathies displays two major bands at 60 and 64 kDa that correspond to tau isoforms lacking exon 10 encoded sequences (3R tau). This profile is found in Pick's disease and FDTP-17. Lastly, in Class IV tauopathies, a major band at 60 kDa and two minor bands at 64 and 69 kDa are found that correspond to tau isoforms devoid of sequences encoded by exons 2, 3, and 10. This class comprises type I and type II myotonic dystrophy.

While many familial tauopathies are caused by mutations in the* MAPT* gene [154], other forms are caused by specific environmental factors. In particular, tau aggregations are found in chronic traumatic encephalopathy (CTE), a neurodegenerative disease first described in boxers and termed dementia pugilistica. In this case, the etiology of the disease is linked to repetitive blast and/or injury to the brain. CTE is characterized by progressive neurodegeneration with widespread deposition of hyperphosphorylated tau as NFTs.

Brain trauma is thought to induce the dissociation of tau from microtubules* via* mechanisms such as intracellular calcium influx, glutamate receptor-mediated excitotoxicity, and kinase activation, leading to hyperphosphorylation of intracellular tau [155–157]. In addition, a sporadic tauopathy, endemic to the island of Guadeloupe, has been linked to the consumption of annonacin, a naturally occurring toxin with mitochondrial complex I inhibitor properties [158]. Low nanomolar concentrations of the toxin have been shown to cause neurodegeneration and induce redistribution of tau from axons to the somatodendritic compartment in neuronal culture [159].* In vivo*, the toxin causes an increase in both tau levels and tau phosphorylation. This toxin also increases neuronal somatodendritic accumulation of hyperphosphorylated tau in transgenic tau^R406W^ mice following a brief 3-day exposure [160].

Initial causative events and precise pathological processes remain largely elusive in tauopathies. Mutations in the* MAPT* gene have been identified in a large number of rare familial tauopathies, but environmental factors such as injuries and toxins, most of which remain to be identified, are also suspected to play a role in the far more frequent sporadic forms of the disease. In particular, multiple lines of evidence point to OS as an important agent in the pathophysiology of neurodegenerative tauopathies.

## 6. Oxidative Stress Is a Common Feature of the Pathophysiology of Neurodegenerative Diseases

Reactive oxygen species (ROS) are chemically reactive molecules containing oxygen, such as superoxide anion (O_2_^−^), hydroperoxyl radical (HO_2_), hydrogen peroxide (H_2_O_2_), and hydroxyl radical (OH^−^). Under normal conditions, cells permanently produce limited amounts of ROS by multiple biochemical processes. The “professional” ROS producers, such as mitochondria, peroxisomes, and endoplasmic reticulum, are the major sources. In mitochondria, ROS are produced permanently as a byproduct of ATP production by the electron transport chain. It is well documented that elevated levels of ROS are highly toxic to cells, in which they damage essential macromolecules, such as DNA, RNA, proteins, and membrane lipids. Importantly, it has been proposed that accumulation of ROS and oxidative damage on macromolecules contribute not only to the physiology of ageing, but also to the pathophysiology of many diseases, including various types of neurodegenerative disorders.

Cells have developed several strategies to manage ROS. In particular, they synthesize several enzymes that display antioxidant properties. Among this large family of antioxidant proteins are catalase, glutathione peroxidase (GPx), and superoxide dismutase (SOD), which protect against the damaging effects of reactive oxygen species (ROS). In particular, the chief defense against superoxide anion produced in the course of respiration is mitochondrial SOD2. However, ROS detoxification is not 100% efficient, and residual superoxides and peroxides persist in cells. In addition, it has been shown that microglial cells produce ROS, either in an attempt to eliminate pathogens or during the first steps of neuroinflammatory processes. Several recent studies have established a direct, albeit complex relationship between neurodegenerative diseases of the AD/tauopathy types and microglial activation [49, 161, 162]. However, this novel and very interesting field of investigation lies outside the scope of this review.

Thus while controlled amounts of ROS are permanently made as a byproduct of cellular metabolism and inflammatory processes under physiological conditions, a pool of antioxidant molecules is permanently produced to detoxify toxic oxygen species. In this context, oxidative stress refers to an imbalance between ROS levels and available antioxidant molecules. In particular, it is well established that sustained oxidative stress can be very damaging to cells, such conditions leading to cell apoptosis as the consequence of activation of the JNK pathway [163, 164].

Several lines of evidence suggest that the balance between ROS and antioxidant defenses is particularly fragile in brain neurons. More importantly, perturbations of this delicate equilibrium are suspected to play a crucial role in many neurodegenerative diseases, first in Alzheimer's disease and more recently in other tauopathies. We know that neuronal cells display unique features and properties: they are permanently postmitotic cells that also show high energy and oxygen consumption, suggesting an elevated rate of ROS production. Brain neurons also contain high levels of transition metals, which can adopt at least two oxidation states, including a reactive one. Iron and copper are tightly linked with the cellular redox state. Fe^2+^ can generate the hydroxyl radical through the fenton reaction [165]. Moreover, the brain has low levels of antioxidants relative to other organs [166].

## 7. Brain Neurons Undergo Oxidative Stress in Tauopathies

A large body of evidence indicates that A*β* deposition in both AD patients and transgenic animal models is associated with an accumulation of OS markers. Similarly, 20 years ago, the first evidence for OS was detected in Pick's disease and CBD patients. Increased levels of heme oxygenase-1 (HO-1), a putative marker of oxidative injury, were detected in Pick bodies in Pick's disease patients and neuropil threads and glial inclusions in patients with CBD [167]. More recently, OS was also found to be associated with tauopathies of the FTLD spectrum of disorders [168] and PSP types [169]. Several OS markers are notably increased in these tauopathies, such as malondialdehyde (MDA), which is formed following the reaction of ROS on polyunsaturated lipids, or 4-hydroxynonenal (HNE) and thiobarbituric acid reactive substances (TBARS) produced by peroxidation of intracellular lipids [168, 170, 171]. Oxidative damage of the glycolytic enzymes fructose bisphosphate aldolase A (aldolase A) and phosphoglycerate kinase-1 (PGK-1) was also detected in the frontal cortex in PSP cases [168].

In addition to OS markers, several studies have reported an activation of antioxidant defenses in several tauopathies. In particular, in several PSP patients, an increased level of SOD2 was detected in the subthalamic nucleus [172], and high levels of Cu/Zn-SOD were found in cerebrospinal fluid [173]. Interestingly, increased levels of SOD, Glutathione peroxidase (GPx), and HNE-conjugated GPx were found in PSP patients that correlated positively with age and thus disease progression [173].

The overexpression of both OS markers and antioxidant enzymes in both patients with different types of tauopathies and animal models strongly suggests that OS is a key component of the pathophysiology of these diseases. However, these data also raise the important question of the role of OS in tau pathologies, a fundamental issue for the development of efficient therapeutic strategies. In other words, is OS an early causal factor in the pathophysiological process or is it merely a consequence of the different cell injuries induced by tau hyperphosphorylation.

## 8. Oxidative Stress, an Early Marker of Tauopathies

Several studies performed on various cellular or animal models of tauopathies have established that overexpression of mutant forms of human tau underlying various types of dominantly inherited tauopathies increases both the expression of OS markers and the sensitivity of neurons to oxidant molecules, such as H_2_O_2_ or paraquat. Following overexpression of wild-type tau in N2a neuroblastoma cells, an increased susceptibility to H_2_O_2_ was observed, linked to peroxisome depletion in neurites due to inhibition of transportation along microtubules [57]. More recently, a proteomic analysis of transgenic mice carrying human tau^P301L^ protein underlying FTDP-17 identified proteins involved in mitochondrial respiration and metabolism (mainly components of respiratory complex V) and antioxidant enzymes (peroxiredoxins 5 and 6, glutathione S-transferase, and GPx) as the two major classes of downregulated proteins. Furthermore, biochemical analysis of these mice showed an increased ROS production and lipid peroxidation in the brain, elevated activities of antioxidant enzymes, and evidence of mitochondrial dysfunction. In this model, increased ROS levels were already detected in 12-month-old tau^P301L^ mice but were more pronounced and statistically significant in 24-month-old individuals, correlating with the age-specific increase in tau pathology [174]. This progressive increase in ROS that parallels the progression of the disease suggests that ROS production is a mere consequence of the pathophysiological process.

However, other studies suggest that oxidative stress is present at earlier stages of the pathologic process in AD [175, 176], and more recently in other tauopathies [177]. Accumulation of a truncated tau fragment has been described in sporadic AD cases, and cultured cortical neurons from a transgenic rat model expressing this truncated protein displayed high levels of OS markers and an increased susceptibility to ROS, resulting in a dramatic increase in mortality upon exposure [178]. These findings suggest that truncation of tau is an early event that precedes OS in this model.

OS and mitochondrial dysfunction accompanied by behavioral deficits were detected prior to tau hyperphosphorylation and NFT accumulation in transgenic mice expressing human tau^P301S^ protein, which underlies another autosomal dominant form of FTDP-17 [177]. In this model, a decreased expression of MnSOD was detected from 7 months of age onward, while tau hyperphosphorylation and tangle formation were only detected 3 months later and were associated with GSK3*β* activation. In these mice also, at 7 and 10 months of age, elevated protein carbonyl levels (an indicator of protein oxidation) were detected in the cerebral cortex, suggesting that ROS production occurs early in the disease process. Importantly, while markers of lipid peroxidation and protein oxidation were markedly elevated at 12 and 24 months of age, ROS levels in isolated brain cortex mitochondria were only mildly increased at the same stages. This result indicates that mitochondria are not the major source of ROS in these transgenic tau^P301S^ mice [177].

In a recent study, several lines of evidence suggest that OS is a primary and causal player in the neurotoxicity induced by tau mutations, through induction of both apoptosis and dysregulated cell cycle activation [179]. First, using a* Drosophila* model that overexpresses human mutant tau^R406W^ associated with FTD, Dias-Santagata et al. [179] showed that genetic downregulation of the antioxidant enzymes SOD2 or thioredoxin reductase (Trxr) increases neurodegenerative phenotypes induced by tau^R406W^ expression. In close agreement with these data, treatment of tau^R406W^ flies with 30 mM paraquat leads to an increased mortality rate, underlining the elevated sensitivity of these flies to OS. Next, using a transgenic line expressing another tau construct, designated tau^E14^, in which 14 S/T phosphorylation sites were mutated to glutamate to mimic phosphorylation, it was shown that flies overexpressing tau^E14^ and heterozygous for either* Sod2* ^*n283*^ or* Trxr* ^Δ*1*^ mutations displayed an increased number of apoptotic cells. Importantly, in these flies, levels of tau hyperphosphorylation were not modified by the downregulation of the antioxidant enzymes. These results clearly show that heterozygous* Sod* or* Trxr* mutations enhance the toxicity of tau^E14^ independently of tau phosphorylation levels. As the JNK signaling pathway is one of the best characterized responses to oxidative damage [163, 180], the authors investigated JNK activation in tau^E14^ individuals and tau^E14^ flies heterozygous for either* Sod2* ^*n283*^ or* Trxr* ^Δ*1*^. Interestingly, compared with levels seen in tau^E14^ individuals, a 6- or 9-fold increased activation of the JNK pathway was detected in tau^E14^ flies heterozygous for either* Sod2* ^*n283*^ or* Trxr* ^Δ*1*^, respectively, suggesting that JNK pathway activity induced by ROS leads to induction of apoptosis. Moreover, in these flies, activation of the JNK pathway was only detected in brain neurons, the sole brain cell type showing significant levels of apoptosis. Finally, while proliferating brain neurons are extremely uncommon in adult wild-type flies, approximately 40 and 100 PCNA and phosphorylated histone 3 (PH3) expressing neurons were detected, in tau^R406W^ individuals and tau^R406W^ flies heterozygous for either* Sod2* ^*n283*^ or* Trxr* ^Δ*1*^, respectively. Thus this study identified both activation of JNK pathway-mediated apoptosis and cell cycle dysregulation as pathogenic processes directly induced by ROS produced as a consequence of the pathophysiological processes induced by tau hyperphosphorylation.

Altogether, these studies identify OS as an early cellular dysfunction in the pathophysiology of tauopathies, which induces both JNK-mediated apoptosis and a dysregulated cell cycle in brain neurons. These data also suggest that either tau hyperphosphorylation* per se,* microtubule disorganization, or some other not yet identified consequences of abnormal tau accumulation promote ROS production and thereby OS in brain neurons. However, the source of ROS in the different models used, that is, mitochondria versus other producers such as microglia, and the precise role of oxidant molecules in the pathologic processes are two key issues that remain unresolved.

## 9. Oxidative Stress Can Promote Tau Hyperphosphorylation and Aggregation

Although the results described above clearly establish that expression of tau mutants underlying tauopathies in either human patients or animal models induces OS in neurons, another line of evidence also suggests that accumulation of ROS can directly stimulate tau hyperphosphorylation and aggregation.

Several studies showed that OS leads to increased tau phosphorylation in neuronal cultures [181, 182]. Moreover, carbonyl-4HNE facilitates aggregation of phosphorylated tau* in vitro* [183] and induces tau hyperphosphorylation [184, 185]. Oxidative events also produce oxidized fatty acids, which have been shown to stimulate tau polymerization* in vitro* [186]. Moreover, in mice deficient in mitochondrial SOD2, an increased tau hyperphosphorylation parallels mitochondrial dysfunction and OS [187]. More recently, because folate deficiency has been linked to neurological disorders [188], a transgenic zebrafish line deficient in folate was produced through overexpression of *γ*-glutamylhydrolase (*γ*-GH), an enzyme that converts active polyglutamyl folates into inactive monoglutamyl folates. Interestingly, this zebrafish transgenic line develops OS associated with tau hyperphosphorylation and aggregation and A*β* plaque formation [189].

However, the mechanism by which OS affects tau phosphorylation remains controversial. In rabbits, clear evidence of mitochondrial injury, OS, increased levels of tau phosphorylation and nuclear translocation of GSK3*β* was observed following intracisternal injection of aggregated A*β*42. Moreover, in the same model, treatment with lithium chloride (LiCl), an inhibitor of GSK3*β*, did not prevent mitochondrial DNA damage or tau hyperphosphorylation, suggesting that the translocation of GSK3*β* may represent an additional event unrelated to tau hyperphosphorylation [190]. GSK3*β* has been identified as one of the major serine/threonine kinases involved in tau phosphorylation/hyperphosphorylation. It was shown that treatment of primary rat cortical neuron cultures with cuprizone, a copper chelator, in combination with oxidant agents Fe^2+^ and H_2_O_2_, significantly increased GSK3*β* activity and pathologic tau hyperphosphorylation. By contrast, concomitant treatment of these cultures with LiCl significantly decreased GSK3*β* activity and reduced abnormal tau phosphorylation, identifying GSK3*β* as the kinase involved in tau phosphorylation following OS conditions in this model [191]. More recently, it was shown that, in neuronal PC12 cells cultured with 100 *μ*M H_2_O_2_, treatment with low doses of GSK3*β* inhibitors protected the cells against H_2_O_2_-induced OS and apoptosis. By contrast, higher concentrations of GSK3*β* inhibitors induce opposite effects relative to apoptosis and tau phosphorylation, demonstrating the key role, albeit ill defined, of this kinase in the disease process. Taken together, these data suggest that GSK3*β* plays important roles in tau pathologies, and fine modulations of its activity may prevent apoptosis, as well as tau phosphorylation, induced by OS [192].

However, beside GSK3*β*, OS affects other signaling pathways and/or kinases mediating tau hyperphosphorylation. In particular, several tau kinases belong to the family of stress-activated protein kinases, which are activated in response to OS [193, 194]. In particular, it has also been shown that HNE directly activates two members of the stress-activated kinase family, JNK and p38, in NT_2_ neuronal cells [195].

Another possible link between OS and pathologic tau phosphorylation is peptidyl prolyl* cis*-trans isomerase 1 (PPIase1) or Pin1. It has been shown that this enzyme is significantly downregulated and oxidized in AD hippocampus. Because Pin1 has been implicated in dephosphorylation of tau protein, it can be hypothesized that* in vivo* oxidative modifications of Pin1, as found in AD hippocampus, reduce Pin1 activity, leading to increased tau phosphorylation [196].

Insulin may also play a role in OS-induced tau phosphorylation. First, it has been established that OS conditions lead to decreased insulin secretion and sensitivity [197, 198]. Second, while insulin is highly sensitive to OS, it plays an important regulatory role in tau phosphorylation in neuronal cell cultures [199, 200], and abnormal insulin levels in mice lead to tau hyperphosphorylation in brain neurons [201, 202].

Thus, whereas accumulation of toxic hyperphosphorylated tau species has been shown to stimulate the production of ROS and thus OS conditions, these data strongly suggest that, in turn, OS directly promotes tau hyperphosphorylation. In this context, tau hyperphosphorylation and OS appear as two elements of a crucial “vicious circle” leading to a progressive coordinated increase in both ROS and abnormal tau and ultimately to cell death.

## 10. Antioxidants in Therapeutic Approaches

Following up the OS theories of aging and neurodegeneration, several antioxidant substances have been tested in different models of tauopathies, showing interesting therapeutic properties at least in these models. In mice overexpressing the smallest human tau isoform, which develop age-dependent filamentous tau inclusions accompanied by neuronal loss and behavioral alterations, dietary supplementation of vitamin E reduced mortality, decreased the number of tau-containing inclusions in the spinal cord, and improved behavioral phenotypes [203]. In another study using a cell model of tauopathy induced by expression of a truncated tau fragment, treatment with the antioxidants vitamin C or vitamin E significantly decreased ROS production [204]. Curcumin, a naturally occurring substance found in turmeric (*Curcuma longa*), is another antioxidant displaying an interesting therapeutic potential. Treatment with curcumin decreased A*β*-induced tau hyperphosphorylation in PC12 cells [205] and okadaic acid-induced ROS production and tau hyperphosphorylation in mice [206]. In addition, in mice lacking superoxide dismutase, antioxidant treatment with catalytic antioxidant EUK189 decreases accumulation of OS markers and tau hyperphosphorylation [187]. Antioxidant therapy has also been shown to inhibit the progression of tau pathology in 3xTg-AD mice, an aggressive mouse model of AD [207]. In these mice, treatment with EUK-207 (a superoxide dismutase/catalase mimetic) from 4 to 9 months of age, that is, before the onset of symptoms, markedly ameliorates disease phenotypes [207]. Similarly, after chronic administration of antioxidant coenzyme Q10 to tau^P301S^ mice, lipid peroxidation was reduced, and survival and behavioral deficits were markedly improved [208]. Also, treatment of these mice with the antioxidant methylene blue diminished oxidative damage, such as oxidized nucleic acids, and tau hyperphosphorylation [209]. Lastly, treatment with vitamin E or overexpression of antioxidant enzyme thioredoxin peroxidase markedly ameliorated the neurodegenerative phenotype of tau^R406W^ transgenic flies [179].

Although much evidence points to antioxidant substances as potential therapeutic agents for the treatment of neurodegenerative diseases of the AD and tauopathy types, translation of the results obtained in animal models into clinical therapeutic strategies has not yet led to significant advances. Hence a better understanding of the role of OS in these diseases is essential, and antioxidant strategies hold promise for slowing down or halting the progression of neurodegenerative tauopathies.

## 11. Conclusion

Both OS and tau hyperphosphorylation appear as key elements in the pathophysiology of tauopathies. However, the relationship between intracellular ROS and tau hyperphosphorylation remains unclear. Accumulation of hyperphosphorylated tau has been shown to cause OS, but ROS have also been shown to stimulate tau hyperphosphorylation. In this context, a better understanding of the role of OS in these pathologies may serve primarily to define novel markers of early stages of the disease and then to develop therapeutic strategies to attenuate, halt, or reverse disease progression. In addition, close interplay between tau hyperphosphorylation and OS suggests that these events are two key components of a vicious circle that plays a crucial role in the pathologic process in tau pathologies, including AD.

## Figures and Tables

**Table 1:** Major pathogenic tau mutations.

Mutation	Type	Effect	Reference
P301L	Substitution	Neuronal impairment, NFT formation	Vogelsberg-Ragaglia, et al., 2000 [94]
P301S	Substitution	Tau hyperphosphorylation and aggregation	Bugiani et al., 1999 [95]
ΔK280	Deletion	Tau aggregation and decreased microtubule affinity, neural loss	Vogelsberg-Ragaglia, et al., 2000 [94]
VPR triple mutant (R406W, P301L, and V337M)	Substitution	Tau hyperphosphorylation, aggregation, and decreased microtubule affinity	Tatebayashi, et al., 2002 [96] Tanemura, et al., 2002 [97]
N296N	Silent	Greater 4R to 3R ratio (splicing)	Spillantini, et al., 2000 [98]

## References

[1] Spillantini M. G., Goedert M. (2013). Tau pathology and neurodegeneration. *The Lancet Neurology*.

[2] Cho J.-H., Johnson G. V. W. (2003). Glycogen synthase kinase 3*β* phosphorylates tau at both primed and unprimed sites: differential impact on microtubule binding. *The Journal of Biological Chemistry*.

[3] Alonso A. D. C., Zaidi T., Novak M., Grundke-Iqbal I., Iqbal K. (2001). Hyperphosphorylation induces self-assembly of *τ* into tangles of paired helical filaments/straight filaments. *Proceedings of the National Academy of Sciences of the United States of America*.

[4] Borza L. R. (2014). A review on the cause-effect relationship between oxidative stress and toxic proteins in the pathogenesis of neurodegenerative diseases. *Revista Medico-Chirurgicala a Societatii de Medici si Naturalisti din Iasi*.

[5] Butterfield D. A., Swomley A. M., Sultana R. (2013). Amyloid *β*-peptide (1–42)-induced oxidative stress in Alzheimer disease: importance in disease pathogenesis and progression. *Antioxidants & Redox Signaling*.

[6] Axelsen P. H., Komatsu H., Murray I. V. (2011). Oxidative stress and cell membranes in the pathogenesis of Alzheimer's disease. *Physiology (Bethesda)*.

[7] Binder L. I., Frankfurter A., Rebhun L. I. (1985). The distribution of tau in the mammalian central nervous system. *Journal of Cell Biology*.

[8] Jeganathan S., Von Bergen M., Mandelkow E.-M. (2008). The natively unfolded character of Tau and its aggregation to Alzheimer-like paired helical filaments. *Biochemistry*.

[9] Kempf M., Clement A., Faissner A., Lee G., Brandt R. (1996). Tau binds to the distal axon early in development of polarity in a microtubule- and microfilament-dependent manner. *The Journal of Neuroscience*.

[10] Tashiro K., Hasegawa M., Ihara Y., Iwatsubo T. (1997). Somatodendritic localization of phosphorylated tau in neonatal and adult rat cerebral cortex. *NeuroReport*.

[11] Loomis P. A., Howard T. H., Castleberry R. P., Binder L. I. (1990). Identification of nuclear tau isoforms in human neuroblastoma cells. *Proceedings of the National Academy of Sciences of the United States of America*.

[12] Klein C., Kramer E.-M., Cardine A.-M., Schraven B., Brandt R., Trotter J. (2002). Process outgrowth of oligodendrocytes is promoted by interaction of Fyn kinase with the cytoskeletal protein Tau. *The Journal of Neuroscience*.

[13] Goedert M., Spillantini M. G., Potier M. C., Ulrich J., Crowther R. A. (1989). Cloning and sequencing of the cDNA encoding an isoform of microtubule-associated protein tau containing four tandem repeats: differential expression of tau protein mRNAs in human brain. *The EMBO Journal*.

[14] Neve R. L., Harris P., Kosik K. S., Kurnit D. M., Donlon T. A. (1986). Identification of cDNA clones for the human microtubule-associated protein tau and chromosomal localization of the genes for tau and microtubule-associated protein 2. *Brain Research*.

[15] Hong M., Zhukareva V., Vogelsberg-Ragaglia V. (1998). Mutation-specific functional impairments in distinct tau isoforms of hereditary FTDP-17. *Science*.

[16] Goedert M., Jakes R. (1990). Expression of separate isoforms of human tau protein: correlation with the tau pattern in brain and effects on tubulin polymerization. *The EMBO Journal*.

[17] Couchie D., Mavilia C., Georgieff I. S., Liem R. K. H., Shelanski M. L., Nunez J. (1992). Primary structure of high molecular weight tau present in the peripheral nervous system. *Proceedings of the National Academy of Sciences of the United States of America*.

[18] Jovanov-Milošević N., Petrović D., Sedmak G., Vukšić M., Hof P. R., Šimić G. (2012). Human fetal tau protein isoform: possibilities for Alzheimer's disease treatment. *International Journal of Biochemistry and Cell Biology*.

[19] Shane Arnold C., Johnson G. V. W., Cole R. N., Dong D. L.-Y., Lee M., Hart G. W. (1996). The microtubule-associated protein tau is extensively modified with *O*-linked *N*-acetylglucosamine. *The Journal of Biological Chemistry*.

[20] Cripps D., Thomas S. N., Jeng Y., Yang F., Davies P., Yang A. J. (2006). Alzheimer disease-specific conformation of hyperphosphorylated paired helical filament-Tau is polyubiquitinated through Lys-48, Lys-11, and Lys-6 ubiquitin conjugation. *The Journal of Biological Chemistry*.

[21] Dorval V., Fraser P. E. (2006). Small ubiquitin-like modifier (SUMO) modification of natively unfolded proteins tau and alpha-synuclein. *The Journal of Biological Chemistry*.

[22] Horiguchi T., Uryu K., Giasson B. I. (2003). Nitration of tau protein is linked to neurodegeneration in tauopathies. *The American Journal of Pathology*.

[23] Ledesma M. D., Bonay P., Avila J. (1995). *τ* Protein from Alzheimer's disease patients is glycated at its tubulin-binding domain. *The Journal of Neurochemistry*.

[24] Cohen T. J., Guo J. L., Hurtado D. E. (2011). The acetylation of tau inhibits its function and promotes pathological tau aggregation. *Nature Communications*.

[25] Wilhelmus M. M. M., Grunberg S. C. S., Bol J. G. J. M. (2009). Transglutaminases and transglutaminase-catalyzed cross-links colocalize with the pathological lesions in Alzheimer's disease brain. *Brain Pathology*.

[26] Miyasaka T., Watanabe A., Saito Y. (2005). Visualization of newly deposited tau in neurofibrillary tangles and neuropil threads. *Journal of Neuropathology & Experimental Neurology*.

[27] Gamblin T. C., Chen F., Zambrano A. (2003). Caspase cleavage of tau: linking amyloid and neurofibrillary tangles in Alzheimer's disease. *Proceedings of the National Academy of Sciences of the United States of America*.

[28] Yuzwa S. A., Macauley M. S., Heinonen J. E. (2008). A potent mechanism-inspired O-GlcNAcase inhibitor that blocks phosphorylation of tau *in vivo*. *Nature Chemical Biology*.

[29] Liu F., Shi J., Tanimukai H. (2009). Reduced O-GlcNAcylation links lower brain glucose metabolism and tau pathology in Alzheimer's disease. *Brain*.

[30] Irwin D. J., Cohen T. J., Grossman M. (2012). Acetylated tau, a novel pathological signature in Alzheimer's disease and other tauopathies. *Brain*.

[31] Nogales E. (2001). Structural insights into microtubule function. *Annual Review of Biophysics and Biomolecular Structure*.

[32] Mukrasch M. D., Biernat J., von Bergen M., Griesinger C., Mandelkow E., Zweckstetter M. (2005). Sites of tau important for aggregation populate *β*-structure and bind to microtubules and polyanions. *The Journal of Biological Chemistry*.

[33] Al-Bassam J., Ozer R. S., Safer D., Halpain S., Milligan R. A. (2002). MAP2 and tau bind longitudinally along the outer ridges of microtubule protofilaments. *The Journal of Cell Biology*.

[34] Fanara P., Husted K. H., Selle K. (2010). Changes in microtubule turnover accompany synaptic plasticity and memory formation in response to contextual fear conditioning in mice. *Neuroscience*.

[35] Qiang L., Yu W., Andreadis A., Luo M., Baas P. W. (2006). Tau protects microtubules in the axon from severing by katanin. *Journal of Neuroscience*.

[36] Yu J.-Z., Rasenick M. M. (2006). Tau associates with actin in differentiating PC12 cells. *The FASEB Journal*.

[37] Takashima A., Murayama M., Murayama O. (1998). Presenilin 1 associates with glycogen synthase kinase-3*β* and its substrate tau. *Proceedings of the National Academy of Sciences of the United States of America*.

[38] Sontag E., Nunbhakdi-Craig V., Lee G. (1999). Molecular interactions among protein phosphatase 2A, tau, and microtubules. Implications for the regulation of tau phosphorylation and the development of tauopathies. *The Journal of Biological Chemistry*.

[39] Kampers T., Friedhoff P., Biernat J., Mandelkow E.-M. (1996). RNA stimulates aggregation of microtubule-associated protein tau into Alzheimer-like paired helical filaments. *FEBS Letters*.

[40] Li W., Wang X. S., Hua Qu M., Liu Y., He R. Q. (2005). Human protein tau represses DNA replication in vitro. *Biochimica et Biophysica Acta*.

[41] Lee G. (2005). Tau and src family tyrosine kinases. *Biochimica et Biophysica Acta: Molecular Basis of Disease*.

[42] Sharma V. M., Litersky J. M., Bhaskar K., Lee G. (2007). Tau impacts on growth-factor-stimulated actin remodeling. *Journal of Cell Science*.

[43] Reynolds C. H., Garwood C. J., Wray S. (2008). Phosphorylation regulates tau interactions with Src homology 3 domains of phosphatidylinositol 3-kinase, phospholipase C*γ*1, Grb2, and Src family kinases. *The Journal of Biological Chemistry*.

[44] Maas T., Eidenmüller J., Brandt R. (2000). Interaction of tau with the neural membrane cortex is regulated by phosphorylation at sites that are modified in paired helical filaments. *The Journal of Biological Chemistry*.

[45] Hwang S. C., Jhon D.-Y., Bae Y. S., Kim J. H., Rhee S. G. (1996). Activation of phospholipase C-*γ* by the concerted action of tau proteins and arachidonic acid. *Journal of Biological Chemistry*.

[46] Perez M., Santa-Maria I., De Barreda E. G. (2009). Tau—an inhibitor of deacetylase HDAC6 function. *Journal of Neurochemistry*.

[47] Yanying M., Jie C., Qipeng Z., Anyang S. (2010). Deletion of *Tau* attenuates heat shock-induced injury in cultured cortical neurons. *Journal of Neuroscience Research*.

[48] Hong X.-P., Peng C.-X., Wei W. (2010). Essential role of tau phosphorylation in adult hippocampal neurogenesis. *Hippocampus*.

[49] Wang L., Jiang Q., Chu J. (2013). Expression of Tau40 induces activation of cultured rat microglial cells. *PLoS ONE*.

[50] Shaw A. S., Filbert E. L. (2009). Scaffold proteins and immune-cell signalling. *Nature Reviews Immunology*.

[51] Dixit R., Ross J. L., Goldman Y. E., Holzbaur E. L. F. (2008). Differential regulation of dynein and kinesin motor proteins by tau. *Science*.

[52] Ebneth A., Godemann R., Stamer K., Illenberger S., Trinczek B., Mandelkow E.-M. (1998). Overexpression of tau protein inhibits kinesin-dependent trafficking of vesicles, mitochondria, and endoplasmic reticulum: implications for Alzheimer's disease. *Journal of Cell Biology*.

[53] Trinczek B., Ebneth A., Mandelkow E.-M., Mandelkow E. (1999). Tau regulates the attachment/detachment but not the speed of motors in microtubule-dependent transport of single vesicles and organelles. *Journal of Cell Science*.

[54] Mandell J. W., Banker G. A. (1996). A spatial gradient of tau protein phosphorylation in nascent axons. *The Journal of Neuroscience*.

[55] Morfini G., Pigino G., Mizuno N., Kikkawa M., Brady S. T. (2007). Tau binding to microtubules does not directly affect microtubule-based vesicle motility. *Journal of Neuroscience Research*.

[56] Yuan A., Kumar A., Peterhoff C., Duff K., Nixon R. A. (2008). Axonal transport rates in vivo are unaffected by tau deletion or overexpression in mice. *The Journal of Neuroscience*.

[57] Stamer K., Vogel R., Thies E., Mandelkow E., Mandelkow E.-M. (2002). Tau blocks traffic of organelles, neurofilaments, and APP vesicles in neurons and enhances oxidative stress. *Journal of Cell Biology*.

[58] Ittner L. M., Fath T., Ke Y. D. (2008). Parkinsonism and impaired axonal transport in a mouse model of frontotemporal dementia. *Proceedings of the National Academy of Sciences of the United States of America*.

[59] Harada A., Oguchi K., Okabe S. (1994). Altered microtubule organization in small-calibre axons of mice lacking tau protein. *Nature*.

[60] Dawson H. N., Ferreira A., Eyster M. V., Ghoshal N., Binder L. I., Vitek M. P. (2001). Inhibition of neuronal maturation in primary hippocampal neurons from tau deficient mice. *Journal of Cell Science*.

[61] Tucker K. L., Meyer M., Barde Y.-A. (2001). Neurotrophins are required for nerve growth during development. *Nature Neuroscience*.

[62] Fujio K., Sato M., Uemura T., Sato T., Sato-Harada R., Harada A. (2007). 14-3-3 Proteins and protein phosphatases are not reduced in tau-deficient mice. *NeuroReport*.

[63] Takei Y., Teng J., Harada A., Hirokawa N. (2000). Defects in axonal elongation and neuronal migration in mice with disrupted tau and map1b genes. *Journal of Cell Biology*.

[64] Ikegami S., Harada A., Hirokawa N. (2000). Muscle weakness, hyperactivity, and impairment in fear conditioning in tau-deficient mice. *Neuroscience Letters*.

[65] Lei P., Ayton S., Finkelstein D. I. (2012). Tau deficiency induces parkinsonism with dementia by impairing APP-mediated iron export. *Nature Medicine*.

[66] Cantero J. L., Moreno-Lopez B., Portillo F., Rubio A., Hita-Yañez E., Avila J. (2011). Role of tau protein on neocortical and hippocampal oscillatory patterns. *Hippocampus*.

[67] Roberson E. D., Scearce-Levie K., Palop J. J. (2007). Reducing endogenous tau ameliorates amyloid *β*-induced deficits in an Alzheimer's disease mouse model. *Science*.

[68] Roberson E. D., Halabisky B., Yoo J. W. (2011). Amyloid-beta/Fyn-induced synaptic, network, and cognitive impairments depend on tau levels in multiple mouse models of Alzheimer's disease. *Journal of Neuroscience*.

[69] Brandt R., Léger J., Lee G. (1995). Interaction of tau with the neural plasma membrane mediated by tau's amino-terminal projection domain. *Journal of Cell Biology*.

[70] Duff K., Knight H., Refolo L. M. (2000). Characterization of pathology in transgenic mice over-expressing human genomic and cDNA tau transgenes. *Neurobiology of Disease*.

[71] Andorfer C., Kress Y., Espinoza M. (2003). Hyperphosphorylation and aggregation of tau in mice expressing normal human tau isoforms. *Journal of Neurochemistry*.

[72] Andorfer C., Acker C. M., Kress Y., Hof P. R., Duff K., Davies P. (2005). Cell-cycle reentry and cell death in transgenic mice expressing nonmutant human tau isoforms. *The Journal of Neuroscience*.

[73] Polydoro M., Acker C. M., Duff K., Castillo P. E., Davies P. (2009). Age-dependent impairment of cognitive and synaptic function in the htau mouse model of Tau pathology. *Journal of Neuroscience*.

[74] Sennvik K., Boekhoorn K., Lasrado R. (2007). Tau-4R suppresses proliferation and promotes neuronal differentiation in the hippocampus of tau knockin/knockout mice. *The FASEB Journal*.

[75] Götz J. (2001). Tau and transgenic animal models. *Brain Research Reviews*.

[76] Alonso A. C., Grundke-Iqbal I., Iqbal K. (1996). Alzheimer's disease hyperphosphorylated tau sequesters normal tau into tangles of filaments and disassembles microtubules. *Nature Medicine*.

[77] Mandelkow E. M., Stamer K., Vogel R., Thies E. (2003). Clogging of axons by tau, inhibition of axonal traffic and starvation of synapses. *Neurobiology of Aging*.

[78] Cuchillo-Ibanez I., Seereeram A., Byers H. L. (2008). Phosphorylation of tau regulates its axonal transport by controlling its binding to kinesin. *The FASEB Journal*.

[79] Ramsden M., Kotilinek L., Forster C. (2005). Age-dependent neurofibrillary tangle formation, neuron loss, and memory impairment in a mouse model of human tauopathy (P301L). *The Journal of Neuroscience*.

[80] Santacruz K., Lewis J., Spires T. (2005). Tau suppression in a neurodegenerative mouse model improves memory function. *Science*.

[81] Hoover B. R., Reed M. N., Su J. (2010). Tau mislocalization to dendritic spines mediates synaptic dysfunction independently of neurodegeneration. *Neuron*.

[82] Hasegawa M., Smith M. J., Goedert M. (1998). Tau proteins with FTDP-17 mutations have a reduced ability to promote microtubule assembly. *FEBS Letters*.

[83] D'Souza I., Poorkaj P., Hong M. (1999). Missense and silent tau gene mutations cause frontotemporal dementia with parkinsonism-chromosome 17 type, by affecting multiple alternative RNA splicing regulatory elements. *Proceedings of the National Academy of Sciences of the United States of America*.

[84] Hasegawa M., Smith M. J., Iijima M., Tabira T., Goedert M. (1999). FTDP-17 mutations N279K and S305N in tau produce increased splicing of exon 10. *FEBS Letters*.

[85] Pickering-Brown S. M., Baker M., Nonaka T. (2004). Frontotemporal dementia with Pick-type histology associated with Q336R mutation in the tau gene. *Brain*.

[86] Barghorn S., Zheng-Fischhofer Q., Ackmann M. (2000). Structure, microtubule interactions, and paired helical filament aggregation by tau mutants of frontotemporal dementias. *Biochemistry*.

[87] Goedert M., Jakes R., Crowther R. A. (1999). Effects of frontotemporal dementia FTDP-17 mutations on heparin-induced assembly of tau filaments. *FEBS Letters*.

[88] Goedert M., Satumtira S., Jakes R. (2000). Reduced binding of protein phosphatase 2A to tau protein with frontotemporal dementia and parkinsonism linked to chromosome 17 mutations. *Journal of Neurochemistry*.

[89] Wszolek Z. K., Tsuboi Y., Ghetti B., Pickering-Brown S., Baba Y., Cheshire W. P. (2006). Frontotemporal dementia and parkinsonism linked to chromosome 17 (FTDP-17). *Orphanet Journal of Rare Diseases*.

[90] Stanford P. M., Shepherd C. E., Halliday G. M. (2003). Mutations in the tau gene that cause an increase in three repeat tau and frontotemporal dementia. *Brain*.

[91] Grover A., Deurel M., Yen S.-H., Hutton M. (2002). Effects on splicing and protein function of three mutations in codon N296 of tau in vitro. *Neuroscience Letters*.

[92] Rizzu P., Van Swieten J. C., Joosse M. (1999). High prevalence of mutations in the microtubule-associated protein tau in a population study of frontotemporal dementia in the Netherlands. *The American Journal of Human Genetics*.

[93] Yoshida H., Crowther R. A., Goeder M. (2002). Functional effects of tau gene mutations ΔN296 and N296H. *Journal of Neurochemistry*.

[94] Vogelgsberg-Ragaglia V., Bruce J., Richter-Landsberg C. (2000). Distinct FTDP-17 missense mutations in Tau produce Tau aggregates and other pathological phenotypes in transfected CHO cells. *Molecular Biology of the Cell*.

[95] Bugiani O., Murrell J. R., Giaccone G. (1999). Frontotemporal dementia and corticobasal degeneration in a family with a P301S mutation in tau. *Journal of Neuropathology and Experimental Neurology*.

[96] Tatebayashi Y., Miyasaka T., Chui D. H. (2002). Tau filament formation and associative memory deficit in aged mice expressing mutant (R406W) human tau. *Proceedings of the National Academy of Sciences of the United States of America*.

[97] Tanemura K., Murayama M., Akagi T. (2002). Neurodegeneration with tau accumulation in a transgenic mouse expressing V337M human tau. *The Journal of Neuroscience*.

[98] Spillantini M. G., Yoshida H., Rizzini C. (2000). A novel tau mutation (N296N) in familial dementia with swollen achromatic neurons and corticobasal inclusion bodies. *Annals of Neurology*.

[99] Noble W., Hanger D. P., Miller C. C. J., Lovestone S. (2013). The importance of tau phosphorylation for neurodegenerative diseases. *Frontiers in Neurology*.

[100] Brion J.-P., Smith C., Couck A.-M., Gallo J.-M., Anderton B. H. (1993). Developmental changes in *τ* phosphorylation: fetal *τ* is transiently phosphorylated in a manner similar to paired helical filament-*τ* characteristic of Alzheimer's disease. *Journal of Neurochemistry*.

[101] Davis D. R., Anderton B. H., Brion J.-P., Reynolds C. H., Hanger D. P. (1997). Oxidative stress induces dephosphorylation of *τ* in rat brain primary neuronal cultures. *Journal of Neurochemistry*.

[102] Zambrano C. A., Egaña J. T., Núñez M. T., Maccioni R. B., González-Billault C. (2004). Oxidative stress promotes tau dephosphorylation in neuronal cells: the roles of cdk5 and PP1. *Free Radical Biology and Medicine*.

[103] Galas M.-C., Dourlen P., Bégard S. (2006). The peptidylprolyl cis/trans-isomerase Pin1 modulates stress-induced dephosphorylation of Tau in neurons: implication in a pathological mechanism related to Alzheimer disease. *Journal of Biological Chemistry*.

[104] Planel E., Miyasaka T., Launey T. (2004). Alterations in glucose metabolism induce hypothermia leading to tau hyperphosphorylation through differential inhibition of kinase and phosphatase activities: implications for Alzheimer's disease. *Journal of Neuroscience*.

[105] Yanagisawa M., Planel E., Ishiguro K., Fujita S. C. (1999). Starvation induces tau hyperphosphorylation in mouse brain: implications for Alzheimer's disease. *FEBS Letters*.

[106] Goedert M., Spillantini M. G., Cairns N. J., Crowther R. A. (1992). Tau proteins of Alzheimer paired helical filaments: abnormal phosphorylation of all six brain isoforms. *Neuron*.

[107] de Leon M. J., DeSanti S., Zinkowski R. (2006). Longitudinal CSF and MRI biomarkers improve the diagnosis of mild cognitive impairment. *Neurobiology of Aging*.

[108] Fagan A. M., Holtzman D. M. (2010). Cerebrospinal fluid biomarkers of Alzheimer's disease. *Biomarkers in Medicine*.

[109] Noble W., Pooler A. M., Hanger D. P. (2011). Advances in tau-based drug discovery. *Expert Opinion on Drug Discovery*.

[110] Ksiezak-Reding H., Liu W.-K., Yen S.-H. (1992). Phosphate analysis and dephosphorylation of modified tau associated with paired helical filaments. *Brain Research*.

[111] Matsuo E. S., Shin R.-W., Billingsley M. L. (1994). Biopsy-derived adult human brain tau is phosphorylated at many of the same sites as Alzheimer's disease paired helical filament tau. *Neuron*.

[112] Hanger D. P., Anderton B. H., Noble W. (2009). Tau phosphorylation: the therapeutic challenge for neurodegenerative disease. *Trends in Molecular Medicine*.

[113] Pei J. J., Grundke-Iqbal I., Iqbal K., Bogdanovic N., Winblad B., Cowburn R. F. (1998). Accumulation of cyclin-dependent kinase 5 (cdk5) in neurons with early stages of Alzheimer's disease neurofibrillary degeneration. *Brain Research*.

[114] Pei J.-J., Braak E., Braak H. (1999). Distribution of active glycogen synthase kinase 3*β* (GSK-3*β*) in brains staged for Alzheimer disease neurofibrillary changes. *Journal of Neuropathology & Experimental Neurology*.

[115] Noble W., Olm V., Takata K. (2003). Cdk5 is a key factor in tau aggregation and tangle formation in vivo. *Neuron*.

[116] Terwel D., Muyllaert D., Dewachter I. (2008). Amyloid activates GSK-3beta to aggravate neuronal tauopathy in bigenic mice. *The American Journal of Pathology*.

[117] Lebouvier T., Scales T. M. E., Williamson R. (2009). The microtubule-associated protein tau is also phosphorylated on tyrosine. *Journal of Alzheimer's Disease*.

[118] Li T., Hawkes C., Qureshi H. Y., Kar S., Paudel H. K. (2006). Cyclin-dependent protein kinase 5 primes microtubule-associated protein tau site-specifically for glycogen synthase kinase 3*β*. *Biochemistry*.

[119] Woods Y. L., Cohen P., Becker W. (2001). The kinase DYRK phosphorylates protein-synthesis initiation factor elF2B*ε* at Ser539 and the microtubule-associated protein tau at Thr212: potential role for DYRK as a glycogen synthase kinase 3-priming kinase. *Biochemical Journal*.

[120] Sharma P., Sharma M., Amin N. D., Albers R. W., Pant H. C. (1999). Regulation of cyclin-dependent kinase 5 catalytic activity by phosphorylation. *Proceedings of the National Academy of Sciences of the United States of America*.

[121] Zukerberg L. R., Patrick G. N., Nikolic M. (2000). Cables links Cdk5 and c-Abl and facilitates Cdk5 tyrosine phosphorylation, kinase upregulation, and neurite outgrowth. *Neuron*.

[122] Liu F., Grundke-Iqbal I., Iqbal K., Gong C.-X. (2005). Contributions of protein phosphatases PP1, PP2A, PP2B and PP5 to the regulation of tau phosphorylation. *European Journal of Neuroscience*.

[123] Qian W., Shi J., Yin X. (2010). PP2A regulates tau phosphorylation directly and also indirectly via activating GSK-3beta. *Journal of Alzheimer's Disease*.

[124] Gong C. X., Lidsky T., Wegiel J., Zuck L., Grundke-Iqbal I., Iqbal K. (2000). Phosphorylation of microtubule-associated protein tau is regulated by protein phosphatase 2A in mammalian brain. Implications for neurofibrillary degeneration in Alzheimer's disease. *The Journal of Biological Chemistry*.

[125] Gong C.-X., Singh T. J., Grundke-Iqbal I., Iqbal K. (1993). Phosphoprotein phosphatase activities in Alzheimer disease brain. *Journal of Neurochemistry*.

[126] Vogelsberg-Ragaglia V., Schuck T., Trojanowski J. Q., Lee V. M.-Y. (2001). PP2A mRNA expression is quantitatively decreased in Alzheimer's disease hippocampus. *Experimental Neurology*.

[127] Zhang Z., Simpkins J. W. (2010). An okadaic acid-induced model of tauopathy and cognitive deficiency. *Brain Research*.

[128] Costa A. P., Tramontina A. C., Biasibetti R. (2012). Neuroglial alterations in rats submitted to the okadaic acid-induced model of dementia. *Behavioural Brain Research*.

[129] Evans D. B., Rank K. B., Bhattacharya K., Thomsen D. R., Gurney M. E., Sharma S. K. (2000). Tau phosphorylation at serine 396 and serine 404 by human recombinant tau protein kinase II inhibits tau's ability to promote microtubule assembly. *The Journal of Biological Chemistry*.

[130] Khlistunova I., Biernat J., Wang Y. (2006). Inducible expression of tau repeat domain in cell models of tauopathy: aggregation is toxic to cells but can be reversed by inhibitor drugs. *The Journal of Biological Chemistry*.

[131] Mocanu M.-M., Nissen A., Eckermann K. (2008). The potential for beta-structure in the repeat domain of tau protein determines aggregation, synaptic decay, neuronal loss, and coassembly with endogenous Tau in inducible mouse models of tauopathy. *The Journal of Neuroscience*.

[132] Spires-Jones T. L., Kopeikina K. J., Koffie R. M., de Calignon A., Hyman B. T. (2011). Are tangles as toxic as they look?. *Journal of Molecular Neuroscience*.

[133] Gómez-Isla T., Hollister R., West H. (1997). Neuronal loss correlates with but exceeds neurofibrillary tangles in Alzheimer's disease. *Annals of Neurology*.

[134] Reed L. A., Wszolek Z. K., Hutton M. (2001). Phenotypic correlations in FTDP-17. *Neurobiology of Aging*.

[135] Chung C. W., Song Y. H., Kim I. K. (2001). Proapoptotic effects of tau cleavage product generated by caspase-3. *Neurobiology of Disease*.

[136] Lasagna-Reeves C. A., Castillo-Carranza D. L., Guerrero-Muñoz M. J., Jackson G. R., Kayed R. (2010). Preparation and characterization of neurotoxic tau oligomers. *Biochemistry*.

[137] Shulman J. M., Feany M. B. (2003). Genetic modifiers of tauopathy in *Drosophila*. *Genetics*.

[138] Wittmann C. W., Wszolek M. F., Shulman J. M. (2001). Tauopathy in *Drosophila*: neurodegeneration without neurofibrillary tangles. *Science*.

[139] Sydow A., van der Jeugd A., Zheng F. (2011). Tau-induced defects in synaptic plasticity, learning, and memory are reversible in transgenic mice after switching off the toxic Tau mutant. *Journal of Neuroscience*.

[140] Goedert M., Jakes R., Spillantini M. G., Hasegawa M., Smith M. J., Crowther R. A. (1996). Assembly of microtubule-associated protein tau into Alzheimer-like filaments induced by sulphated glycosaminoglycans. *Nature*.

[141] Pérez M., Valpuesta J. M., Medina M., de Garcini E. M., Avila J. (1996). Polymerization of tau into filaments in the presence of heparin: the minimal sequence required for tau-tau interaction. *Journal of Neurochemistry*.

[142] Wilson D. M., Binder L. I. (1997). Free fatty acids stimulate the polymerization of tau and amyloid *β* peptides: in vitro evidence for a common effector of pathogenesis in Alzheimer's disease. *The American Journal of Pathology*.

[143] Braak H., Braak E. (1991). Neuropathological stageing of Alzheimer-related changes. *Acta Neuropathologica*.

[144] Saito Y., Ruberu N. N., Sawabe M. (2004). Staging of argyrophilic grains: an age-associated tauopathy. *Journal of Neuropathology and Experimental Neurology*.

[145] Kfoury N., Holmes B. B., Jiang H., Holtzman D. M., Diamond M. I. (2012). Trans-cellular propagation of tau aggregation by fibrillar species. *The Journal of Biological Chemistry*.

[146] Holmes B. B., DeVos S. L., Kfoury N. (2013). Heparan sulfate proteoglycans mediate internalization and propagation of specific proteopathic seeds. *Proceedings of the National Academy of Sciences of the United States of America*.

[147] Clavaguera F., Bolmont T., Crowther R. A. (2009). Transmission and spreading of tauopathy in transgenic mouse brain. *Nature Cell Biology*.

[148] Iba M., Guo J. L., McBride J. D., Zhang B., Trojanowski J. Q., Lee V. M.-Y. (2013). Synthetic tau fibrils mediate transmission of neurofibrillary tangles in a transgenic mouse model of Alzheimer's-like tauopathy. *The Journal of Neuroscience*.

[149] de Calignon A., Polydoro M., Suárez-Calvet M. (2012). Propagation of tau pathology in a model of early Alzheimer's disease. *Neuron*.

[150] Liu L., Drouet V., Wu J. W. (2012). Trans-synaptic spread of tau pathology in vivo. *PLoS ONE*.

[151] Hasegawa M. (2006). Biochemistry and molecular biology of tauopathies. *Neuropathology*.

[152] Richter-Landsberg C. (2000). The oligodendroglia cytoskeleton in health and disease. *Journal of Neuroscience Research*.

[153] Schraen-Maschke S., Sergeant N., Dhaenens C.-M. (2008). Tau as a biomarker of neurodegenerative diseases. *Biomarkers in Medicine*.

[154] Goedert M., Ghetti B., Spillantini M. G. (2012). Frontotemporal dementia: implications for understanding Alzheimer disease. *Cold Spring Harbor Perspectives in Medicine*.

[155] McKee A. C., Cantu R. C., Nowinski C. J. (2009). Chronic traumatic encephalopathy in athletes: progressive tauopathy after repetitive head injury. *Journal of Neuropathology & Experimental Neurology*.

[156] McKee A. C., Stein T. D., Nowinski C. J. (2013). The spectrum of disease in chronic traumatic encephalopathy. *Brain*.

[157] Omalu B., Bailes J., Hamilton R. L. (2011). Emerging histomorphologic phenotypes of chronic traumatic encephalopathy in american athletes. *Neurosurgery*.

[158] Lannuzel A., Ruberg M., Michel P. P. (2008). Atypical parkinsonism in the Caribbean island of Guadeloupe: etiological role of the mitochondrial complex I inhibitor annonacin. *Movement Disorders*.

[159] Escobar-Khondiker M., Höllerhage M., Muriel M.-P. (2007). Annonacin, a natural mitochondrial complex I inhibitor, causes tau pathology in cultured neurons. *The Journal of Neuroscience*.

[160] Yamada E. S., Respondek G., Müssner S. (2014). Annonacin, a natural lipophilic mitochondrial complex I inhibitor, increases phosphorylation of tau in the brain of FTDP-17 transgenic mice. *Experimental Neurology*.

[161] Yoshiyama Y., Higuchi M., Zhang B. (2007). Synapse loss and microglial activation precede tangles in a P301S tauopathy mouse model. *Neuron*.

[162] Lastres-Becker I., Innamorato N. G., Jaworski T. (2014). Fractalkine activates NRF2/NFE2L2 and heme oxygenase 1 to restrain tauopathy-induced microgliosis. *Brain*.

[163] Martindale J. L., Holbrook N. J. (2002). Cellular response to oxidative stress: signaling for suicide and survival. *Journal of Cellular Physiology*.

[164] Matsuzawa A., Ichijo H. (2005). Stress-responsive protein kinases in redox-regulated apoptosis signaling. *Antioxidants and Redox Signaling*.

[165] Jomova K., Vondrakova D., Lawson M., Valko M. (2010). Metals, oxidative stress and neurodegenerative disorders. *Molecular and Cellular Biochemistry*.

[166] Uttara B., Singh A. V., Zamboni P., Mahajan R. T. (2009). Oxidative stress and neurodegenerative diseases: a review of upstream and downstream antioxidant therapeutic options. *Current Neuropharmacology*.

[167] Castellani R., Smith M. A., Richey P. L., Kalaria R., Gambetti P., Perry G. (1995). Evidence for oxidative stress in Pick disease and corticobasal degeneration. *Brain Research*.

[168] Martínez A., Carmona M., Portero-Otin M., Naudí A., Pamplona R., Ferrer I. (2008). Type-dependent oxidative damage in frontotemporal lobar degeneration: cortical astrocytes are targets of oxidative damage. *Journal of Neuropathology and Experimental Neurology*.

[169] Litvan I. (2004). Update on progressive supranuclear palsy. *Current Neurology and Neuroscience Reports*.

[170] Albers D. S., Augood S. J., Martin D. M., Standaert D. G., Vonsattel J. P. G., Beal M. F. (1999). Evidence for oxidative stress in the subthalamic nucleus in progressive supranuclear palsy. *Journal of Neurochemistry*.

[171] Odetti P., Garibaldi S., Norese R. (2000). Lipoperoxidation is selectively involved in progressive supranuclear palsy. *Journal of Neuropathology & Experimental Neurology*.

[172] Cantuti-Castelvetri I., Keller-McGandy C. E., Albers D. S. (2002). Expression and activity of antioxidants in the brain in progressive supranuclear palsy. *Brain Research*.

[173] Aoyama K., Matsubara K., Kobayashi S. (2006). Aging and oxidative stress in progressive supranuclear palsy. *European Journal of Neurology*.

[174] David D. C., Hauptmann S., Scherping I. (2005). Proteomic and functional analyses reveal a mitochondrial dysfunction in P301L tau transgenic mice. *The Journal of Biological Chemistry*.

[175] Perry G., Nunomura A., Hirai K. (2002). Is oxidative damage the fundamental pathogenic mechanism of Alzheimer's and other neurodegenerative diseases?. *Free Radical Biology and Medicine*.

[176] Smith M. A., Nunomura A., Lee H.-G. (2005). Chronological primacy of oxidative stress in Alzheimer disease. *Neurobiology of Aging*.

[177] Dumont M., Stack C., Elipenahli C. (2011). Behavioral deficit, oxidative stress, and mitochondrial dysfunction precede tau pathology in P301S transgenic mice. *The FASEB Journal*.

[178] Cente M., Filipcik P., Pevalova M., Novak M. (2006). Expression of a truncated tau protein induces oxidative stress in a rodent model of tauopathy. *European Journal of Neuroscience*.

[179] Dias-Santagata D., Fulga T. A., Duttaroy A., Feany M. B. (2007). Oxidative stress mediates tau-induced neurodegeneration in *Drosophila*. *The Journal of Clinical Investigation*.

[180] Zhu X., Raina A. K., Lee H.-G. (2003). Oxidative stress and neuronal adaptation in Alzheimer disease: the role of SAPK pathways. *Antioxidants & Redox Signaling*.

[181] Zhu X., Lee H. G., Casadesus G. (2005). Oxidative imbalance in Alzheimer's disease. *Molecular Neurobiology*.

[182] Su B., Wang X., Lee H.-G. (2010). Chronic oxidative stress causes increased tau phosphorylation in M17 neuroblastoma cells. *Neuroscience Letters*.

[183] Pérez M., Cuadros R., Smith M. A., Perry G., Avila J. (2000). Phosphorylated, but not native, tau protein assembles following reaction with the lipid peroxidation product, 4-hydroxy-2-nonenal. *FEBS Letters*.

[184] Liu Q., Smith M. A., Avilá J. (2005). Alzheimer-specific epitopes of tau represent lipid peroxidation-induced conformations. *Free Radical Biology and Medicine*.

[185] Gómez-Ramos A., Díaz-Nido J., Smith M. A., Perry G., Avila J. (2003). Effect of the lipid peroxidation product acrolein on tau phosphorylation in neural cells. *Journal of Neuroscience Research*.

[186] Gamblin T. C., King M. E., Kuret J., Berry R. W., Binder L. I. (2000). Oxidative regulation of fatty acid-induced tau polymerization. *Biochemistry*.

[187] Melov S., Adlard P. A., Morten K. (2007). Mitochondrial oxidative stress causes hyperphosphorylation of tau. *PLoS ONE*.

[188] Stover P. J. (2009). One-carbon metabolism-genome interactions in folate-associated pathologies. *Journal of Nutrition*.

[189] Kao T. T., Chu C., Lee G. (2014). Folate deficiency-induced oxidative stress contributes to neuropathy in young and aged zebrafish—implication in neural tube defects and Alzheimer's diseases. *Neurobiology of Disease*.

[190] Ghribi O., Herman M. M., Savory J. (2003). Lithium inhibits A*β*-induced stress in endoplasmic reticulum of rabbit hippocampus but does not prevent oxidative damage and tau phosphorylation. *Journal of Neuroscience Research*.

[191] Lovell M. A., Xiong S., Xie C., Davies P., Markesbery W. R. (2004). Induction of hyperphosphorylated tau in primary rat cortical neuron cultures mediated by oxidative stress and glycogen synthase kinase-3. *Journal of Alzheimer's Disease*.

[192] Lee K.-Y., Koh S.-H., Noh M. Y., Park K.-W., Lee Y. J., Kim S. H. (2007). Glycogen synthase kinase-3*β* activity plays very important roles in determining the fate of oxidative stress-inflicted neuronal cells. *Brain Research*.

[193] Goedert M., Hasegawa M., Jakes R., Lawler S., Cuenda A., Cohen P. (1997). Phosphorylation of microtubule-associated protein tau by stress-activated protein kinases. *FEBS Letters*.

[194] Atzori C., Ghetti B., Piva R. (2001). Activation of the JNK/p38 pathway occurs in diseases characterized by tau protein pathology and is related to tau phosphorylation but not to apoptosis. *Journal of Neuropathology and Experimental Neurology*.

[195] Tamagno E., Parola M., Bardini P. (2005). *β*-site APP cleaving enzyme up-regulation induced by 4-hydroxynonenal is mediated by stress-activated protein kinases pathways. *Journal of Neurochemistry*.

[196] Sultana R., Boyd-Kimball D., Poon H. F. (2006). Oxidative modification and down-regulation of Pin1 in Alzheimer's disease hippocampus: a redox proteomics analysis. *Neurobiology of Aging*.

[197] Gerbitz K.-D., Gempel K., Brdiczka D. (1996). Mitochondria and diabetes: genetic, biochemical, and clinical implications of the cellular energy circuit. *Diabetes*.

[198] Moreira P. I., Santos M. S., Seiça R., Oliveira C. R. (2007). Brain mitochondrial dysfunction as a link between Alzheimer's disease and diabetes. *Journal of the Neurological Sciences*.

[199] Lesort M., Jope R. S., Johnson G. V. W. (1999). Insulin transiently increases tau phosphorylation: involvement of glycogen synthase kinase-3*β* and Fyn tyrosine kinase. *Journal of Neurochemistry*.

[200] Lesort M., Johnson G. V. W. (2000). Insulin-like growth factor-1 and insulin mediate transient site-selective increases in tau phosphorylation in primary cortical neurons. *Neuroscience*.

[201] Schubert M., Brazil D. P., Burks D. J. (2003). Insulin receptor substrate-2 deficiency impairs brain growth and promotes tau phosphorylation. *The Journal of Neuroscience*.

[202] Schechter R., Beju D., Miller K. E. (2005). The effect of insulin deficiency on tau and neurofilament in the insulin knockout mouse. *Biochemical and Biophysical Research Communications*.

[203] Nakashima H., Ishihara T., Yokota O. (2004). Effects of *α*-tocopherol on an animal model of tauopathies. *Free Radical Biology and Medicine*.

[204] Cente M., Filipcik P., Mandakova S., Zilka N., Krajciova G., Novak M. (2009). Expression of a truncated human tau protein induces aqueous-phase free radicals in a rat model of tauopathy: implications for targeted antioxidative therapy. *Journal of Alzheimer's Disease*.

[205] Park S.-Y., Kim H.-S., Cho E.-K. (2008). Curcumin protected PC12 cells against beta-amyloid-induced toxicity through the inhibition of oxidative damage and tau hyperphosphorylation. *Food and Chemical Toxicology*.

[206] Rajasekar N., Dwivedi S., Tota S. K. (2013). Neuroprotective effect of curcumin on okadaic acid induced memory impairment in mice. *European Journal of Pharmacology*.

[207] Clausen A., Bi X., Baudry M. (2012). Effects of the superoxide dismutase/catalase mimetic EUK-207 in a mouse model of Alzheimer's disease: protection against and interruption of progression of amyloid and tau pathology and cognitive decline. *Journal of Alzheimer's Disease*.

[208] Elipenahli C., Stack C., Jainuddin S. (2012). Behavioral improvement after chronic administration of coenzyme Q10 in P301S transgenic mice. *Journal of Alzheimer's Disease*.

[209] Stack C., Jainuddin S., Elipenahli C. (2014). Methylene blue upregulates Nrf2/ARE genes and prevents tau-related neurotoxicity. *Human Molecular Genetics*.

